# Exploring Ibuprofen–Menthol Eutectic Systems: Physicochemical Properties and Cytotoxicity for Pharmaceutical Applications

**DOI:** 10.3390/pharmaceutics17080979

**Published:** 2025-07-29

**Authors:** Álvaro Werner, Estefanía Zuriaga, Marina Sanz, Fernando Bergua, Beatriz Giner, Carlos Lafuente, Laura Lomba

**Affiliations:** 1 Facultad de Ciencias de la Salud, Universidad San Jorge, Campus Universitario, Autov. A23 km 299, 50830 Villanueva de Gállego, Zaragoza, Spain; awerner@usj.es (Á.W.); ezuriaga@usj.es (E.Z.); bginer@usj.es (B.G.); 2 Departamento de Química Física, Facultad de Ciencias, Universidad de Zaragoza, 50009 Zaragoza, Zaragoza, Spain; ferber@posta.unizar.es (F.B.); celadi@unizar.es (C.L.)

**Keywords:** physicochemical properties, cytotoxicity, Therapeutic eutectic systems, ibuprofen, menthol

## Abstract

**Backgroun/Objectives:** Recent pharmaceutical research has increasingly focused on eutectic systems to improve the formulation and delivery of active pharmaceutical ingredients (APIs). This study presents the preparation and characterization of three therapeutic eutectic systems (THEESs) based on ibuprofen and menthol at various molar ratios. **Methods:** The THEESs were prepared and analyzed by assessing their physicochemical properties and rheological properties were evaluated to determine flow behavior. Cytotoxicity assays were conducted on HaCaT and HepG2 cell lines to assess biocompatibility. **Results:** All systems formed monophasic, homogeneous, clear and viscous liquids. Key physicochemical properties, including density, refractive index, surface tension, speed of sound and isobaric heat capacity, showed a temperature-dependent, inverse proportional trend. Viscosity followed the Vogel–Fulcher–Tammann equation, and rheological analysis revealed non-Newtonian behavior, which is important for pharmaceutical processing. Notably, cytotoxicity assays revealed that Ibu-M3 and Ibu-M4 showed lower toxicity than pure compounds in HaCaT cells, while Ibu-M5 was more toxic; in HepG2 cells, only Ibu-M3 was less toxic, whereas Ibu-M4 and Ibu-M5 were more cytotoxic than the pure compounds. **Conclusions:** These findings highlight the potential of ibuprofen–menthol eutectic systems for safer and more effective pharmaceutical formulations.

## 1. Introduction

The search for new Deep Eutectic Systems (DESs) in the pharmaceutical field has boomed in recent years [1,2,3]. DESs are combinations of two or more substances that, when mixed in a specific molar proportion, exhibit a melting point much lower than that of each individual counterpart [4,5]. If these systems consist of natural substances, they are called Natural Deep Eutectic Systems (NADESs) [6,7]. If one or more of the system components are an active principle, they are called Therapeutical Deep Eutectic Systems (THEDESs) [8].

In 1998, Stott et al. developed DESs formed by ibuprofen and various terpene molecules (thymol and menthol). The objective of this work was to improve the ability of ibuprofen to penetrate the skin [9]. However, this type of system was named THEDES after the study presented by Aroso et al. where they prepared and analyzed their systems formed by menthol and ibuprofen and developed controlled drug release systems [10]. The same researchers also carried out a study in which different formulations using choline chloride or menthol in combination with various active compounds, including phenylacetic acid, benzoic acid and acetylsalicylic acid, were created and prepared in order to analyze their chemical structure, thermal behavior and antibacterial properties [11].

There are studies involving THEDESs such as ibuprofen and menthol (1:3) or ibuprofen and safranal (1:1, 1:2, 1:3, 1:4 and 1:8), among others, that have been studied before, as in the study conducted by Pereira et al. They prepared the systems, characterized them and conducted studies on solubility and permeability. Furthermore, they investigated the anti-inflammatory potential of these systems through various assays [12]. Pereira et al., in addition, studied systems formed by ibuprofen and limonene (1:8), carrying out their characterization, the toxicity assessment and the evaluation of their antiproliferative effects, solubility and bioactivity [13]. Silva et al. published a THEDES formed by perillyl alcohol and ibuprofen in molar ratios of 4:1, 6:1 and 8:1. The mixtures were characterized, and the solubility and the antimicrobial potential were evaluated [14].

Roda et al. published the creation and preparation of drug formulations formed by citric acid and arginine, using green supercritical CO_2_ technology with the aim of targeting tuberculosis. They developed an innovative inhalable drug delivery system containing encapsulated therapeutic DESs [15].

These examples illustrate the versatility of these mixtures and their promise for the future of pharmaceutical formulation. In fact, this potential is already a reality beyond the academia realm in these other examples; for instance, the European Medicines Agency (EMA) and the Food and Drug Administration (FDA) have already approved various formulations based on THEDESs, such as EMLA^®^ (lidocaine/prilocaine), SYNERA^®^ (lidocaine/tetracaine), PLIAGLIS (lidocaine/tetracaine) or FORTACIN (lidocaine/prilocaine) [5].

Considering the previous information, the main aim of the study is to prepare three Therapeutical Eutectic Systems (THEESs) formed by ibuprofen and menthol in different molar ratios with the aim of reaching a detailed knowledge of their characteristics and prospective applications. The conducted study encompasses a range of characteristics related to the composition, structure and toxicity of these eutectic mixtures.

Ibuprofen, classified as a nonsteroidal anti-inflammatory drug (NSAID), is utilized to decrease fever and treat pain or swelling due to a range of issues like headache, toothache, back pain, arthritis, menstrual cramps or minor injuries [16]. On the other hand, menthol is an organic compound, present in peppermint and various other mint species that can also be chemically synthesized from limonene or mint oils. It is commonly used as a flavoring or fragrance in a variety of products such as toothpaste, mouthwash, cough drops and lip balms [17]. It delivers a sensation of coolness upon skin contact, which can alleviate discomfort in the tissue beneath. Frequently, it is applied externally to offer short-term relief from mild arthritis, back pain, muscle or joint aches and painful bruising [18]. It can also be found in medical products such as creams, ointments, sprays and lotions used to treat infections of the throat [19,20,21].

In this work, the THEESs under study have been studied from a chemical and cytotoxic point of view. Different spectroscopic studies have been carried out to investigate the molecular insights of the mixtures. A Differential Scanning Calorimetry, DSC, has been carried out in order to measure the melting point and different properties, including density, sound velocity, refractive index, viscosity and surface tension, among others, have been measured as well. In addition, given that the rheological behavior of a dosage form plays an important role in determining drug quality and production effectiveness, the studied mixtures were also subjected to rheological assessment [22]. Finally, a cytotoxic study of these systems has been carried out in order to evaluate the effect on two cell lines (tumor and non-tumor) such as HepG2 (liver) and HaCaT (keratinocytes).

## 2. Materials and Methods

### 2.1. Chemicals

Dl-menthol has been supplied by Thermoscientific (Madrid, Spain) with 98% purity and ibuprofen has been obtained from Fagron (Barcelona, Spain) with 98.5% purity.

### 2.2. Preparation of Therapeutical Eutectic Systems

Briefly, 50 mL of each THEES was obtained by the combination of menthol and ibuprofen at different molar ratios. The precise amounts of each pure compound were weighed using a Sartorius Entris 5201-1S balance (Göttingen, Germany) with an accuracy of ±0.1 g. The components were then combined in a container and subjected to constant stirring and heating in a water bath at 60–70 °C until a clear, homogeneous mixture formed. The prepared systems were stored in the dark until needed for further experiments. The following formula was employed to calculate the molar mass of each mixture:

MW_DES_ = X_ibuprofen_·MW_ibuprofen_ + X_menthol_·MW_menthol_ (1)

where X is the mole fraction and MW is the molar mass.

In Table 1, the abbreviation, molar ratio and molecular weight of three studied THEESs are shown.

### 2.3. NMR Spectra

NMR measurements were collected on a Bruker AVANCE spectrometer (Madrid, Spain) operating at 400 MHz and maintained at 300 K. For each ^1^H NMR spectrum, 8 scans were acquired using the zg30 single-pulse sequence, with a spectral width of 16 ppm centered at 5 ppm and a relaxation delay of 3 s. For the ^13^C NMR spectra, 64 scans (using the APT jmod sequence program) with a spectral width of 240 ppm, centered at 110 ppm and a relaxation time of 2 s were acquired. Several routines, such as DQF-COSY, ^1^H–^13^C HSQC and ^1^H–^13^C HMBC (with Bruker pulse programs cosygpmfqf, hsqcedetgp and hmbclpndqf, respectively), were employed for signal assignment. Additionally, NOESY and DOSY experiments were carried out to assess the interactions among the mixture’s components.

### 2.4. Differential Scanning Calorimetry (DSC)

In this work, both the Solid–Liquid Equilibria (SLE) and isobaric heat capacity were investigated utilizing a TA Instruments DSC Q2000 Differential Scanning Calorimeter fitted with an RCS cooling system (New Castle, England). To ensure precise measurements, temperature and heat flow were calibrated against the melting point of indium as a reference standard, revealing uncertainties of 0.5 K in temperature (*T_m_*) and 1% in molar enthalpy change (Δ*_m_H*). For the determination of isobaric heat capacity, an additional calibration using sapphire as a reference standard is required. This calibration was conducted before the first measurement and after the latter one to monitor and mitigate any potential temporal variations in the heat capacity calibration factor. The calculated uncertainty in *c*_p_, compared against established data within the 263–343 K temperature range, was found to be less than 1%. For each mixture, samples ranging from 5 to 15 mg were placed in aluminum pans in their liquid state to guarantee the molar fraction of the sample is identical to that prepared. When the sample was solid at room temperature, a preheating procedure in the DSC was applied to ensure that crystallization took place under consistent conditions for all samples analyzed. The cooling and heating processes were performed at controlled rates, with reported temperatures representing peak values due to thermal anomalies, i.e., presence of pretails. This comprehensive approach ensured the accuracy of the obtained data in studying SLE.

### 2.5. Thermophysical Properties

A range of properties—including density, refractive index, surface tension, speed of sound, isobaric heat capacity and viscosity—have been determined. Measurements were conducted at temperature intervals of 2.5 K across the 278.15 to 338.15 K range. Furthermore, additional properties such as isentropic compressibility were calculated based on the primary experimental results.

The density and speed of sound were concurrently obtained with an Anton Paar DSA 5000 vibrating tube sound analyzer and densimeter (Ostfildern, Germany) operating at a frequency of 3 MHz. To calibrate the equipment, dry air and ultrapure water from SH Calibration Service GmbH (Berlin, Germany) were employed. Temperature control was performed internally, with a precision of ±0.005 K.

The measurement uncertainty was 0.5 m·s^−1^ for the speed of sound and 0.1 kg·m^−3^ for density. The isentropic compressibility, *κ_s_*, can be determined using the following equation:

(2) $$\kappa_{s}=1/(\rho u^{2})$$

assuming that the ultrasonic absorption is zero.

The refractive indices, *n*_D_, at a wavelength of 589.3 nm were determined using the Abbemat-HP DR refractometer by Kernchen (Madrid, Spain). Temperature was regulated internally with a precision of ±0.01 K, leading to a measurement uncertainty of 5 × 10^−5^.

The surface tensions of the investigated systems were determined using a Lauda TVT-2 drop volume tensiometer (Lauda-Königshofen, Germany). Temperature control was achieved using a Lauda E-200 thermostat (Lauda-Königshofen, Germany) with a precision of ±0.01 K. The experimental uncertainty for surface tension measurements was determined to be 0.2 mN·m^−1^. To gain insight into the molecular arrangement at the surface, both the entropy, Δ*S_s_*, and enthalpy, Δ*H_s_*, of surface formation per unit area were calculated using the following equations:

(3) $${\Delta S}_{s}=-{\left(d\sigma /dT\right)}_{P}$$

(4) $${\Delta H}_{s}=\sigma -T{\left(d\sigma /dT\right)}_{P}$$

Kinematic viscosities *ν*, were determinated using a Schott-Geräte AVS-440 system equipped with Ubbelohde capillary viscometers from Schott Instruments GmbH (Mainz, Germany), applying kinetic corrections as needed. The measurement uncertainty was 0.01 s. Temperature was regulated with a Schott-Geräte CT 1150/2 thermostat from Schott Instruments GmbH (Mainz, Germany) ensuring a precision of ±0.01 K. Dynamic viscosity η=ρν, was calculated from the experimentally obtained kinematic viscosity values, with an associated uncertainty of 1%. All measurements were performed in triplicate (*n* = 3).

### 2.6. Rheological Properties

To perform a rheological study, the viscosity of systems being investigated was assessed utilizing Brookfield DV-E rotational viscometer (Middleborough, MA, USA). Shear rate was varied by adjusting the rotational speed from the maximum (typically 60 rpm) down to the minimum (0.3 rpm). Temperature was precisely maintained at 25 °C with an immersion bath (Termotronic JPSELECTA from Thermoscientific (Madrid, Spain)), providing an accuracy of ±0.1 °C. Viscosity measurements were conducted in triplicate to ensure accuracy and reliability. This property was measured three times (*n* = 3) to obtain consistent results.

### 2.7. Cytotoxicity Study

#### 2.7.1. Cell Culture

Due to their suitability for cytotoxicity assessments in in vitro models, two different cell lines were used in this study: HaCaT (normal adult human primary epidermal keratinocytes, non-tumorous) and HepG2 (hepatocyte carcinoma). By utilizing both tumorous and non-tumorous cell lines, this study enhances the comprehensiveness of toxicological evaluations, allowing for the assessment of potential risks associated with substance exposure through different mechanisms.

The cell cultures were maintained using Advanced-DMEMF12 medium supplemented with 10% FBS, 1% glutamine (2 mM) and 1% penicillin and streptomycin. Cell seeding was performed at a density of 6000 cells/cm^2^ in T25 flasks and incubated at 37 °C in a humidified atmosphere with 5% CO_2_ until reaching 90% confluence, after which they were passaged. All reagents required for the experiments were sourced from Fisher Scientific.

#### 2.7.2. Concentrations

Various concentrations of ibuprofen and menthol were created by dissolving these substances in a cell culture medium. The mixtures were meticulously formulated with an MEM10X medium, supplemented with 1% penicillin–streptomycin, 10% Fetal Bovine Serum (FBS), 1X HEPES and ultrapure water. To establish different concentrations, the following levels were employed, 4000 mg/L, 2000 mg/L, 1000 mg/L, 500 mg/L, 250 mg/L and 125 mg/L, for both pure compounds and combination systems. Subsequently, the pH of the solutions was carefully adjusted to pH = 7. Lastly, the stock solutions underwent filtration using a 0.22 μm filter to ensure purity and remove any particulate matter.

#### 2.7.3. Cell Viability Assay

Two separate cell viability assays were performed: the PrestoBlue assay and the crystal violet assay. To ensure robust results, both experiments were carried out in triplicate. For testing different concentrations, 3-well plates were utilized, while 6-well plates were employed for control purposes. This meticulous approach ensured the reliability of the data collected during the assays.

##### Prestoblue Assay

For this test, PrestoBlue cell viability reagent was employed. Initially, cells were seeded (6000 cells per well in 96-well plates) and cultured at 37 °C in a 5% CO_2_ environment for a period of 4 days until they reached confluence. Subsequently, these cells were subjected to a range of concentrations of the DES under investigation. After 24 h of incubation, the cells underwent two washes with phosphate-buffered saline (PBS). Following this, PrestoBlue reagent was added to each well, diluted in the cell culture medium at a ratio of 1/10 and incubated for 1 h at 37 °C. In the final step, fluorescence was measured using a microplate reader (Bio-Tek Synergy H1, Bad Friedrichshall, Germany), with excitation and emission wavelengths set to 530 nm and 590 nm, respectively. This comprehensive process allowed for the assessment of cell viability and experimental outcomes.

##### Crystal Violet Assay

Following a 24 h incubation with the test mixtures, the cells underwent two washes with PBS and were subsequently fixed using 4% paraformaldehyde, incubating for 15 min at room temperature. After fixation, each well was washed with PBS. Subsequently, the cells were treated with a 0.1% crystal violet solution prepared in PBS and allowed to incubate for 30 min at ambient temperature. Any excess dye was carefully removed using tap water, and images were captured using an inverted microscope LEICA from DEMLab (Zaragoza, Spain) for visual assessment.

For precise quantitative measurements, the crystals formed by crystal violet were dissolved using a 10% acetic acid solution. Subsequently, absorbance at 590 nm was measured with a plate reader to assess the concentration of crystal violet. This comprehensive procedure allowed for both qualitative and quantitative analysis of the experimental results.

#### 2.7.4. BCA Protein Assay Kit

After the 24 h incubation with the test compounds in the cell viability assay, the quantification of total protein content per well was carried out using the BCA method. Cells were washed twice with PBS and lysed by adding NaOH 0.1 M to each well and incubated for 20 min at room temperature with agitation. A duplicate BCA protein standard was prepared (0, 50, 100, 400, 600 and 800 μg/mL of albumin) and then 200 μL/well of the BCA protein assay kit working reagent was added and incubated at 37 °C for 30 min. Results were obtained by measuring absorbance at 562 nm with a plate reader. Each mixture concentration was tested in triplicate using 3-well plates, while controls were assessed in 6-well plates to ensure reproducibility.

### 2.8. Statistical Analysis

Using GraphPad Prism version 10.0, statistical comparisons between groups were performed by applying one-way ANOVA, followed by the Tukey–Kramer honestly significant difference post hoc test. The analysis was based on a 95% confidence level, so results with a *p*-value less than 0.05 were considered statistically significant. Under these conditions, the null hypothesis (H0)—which proposes that all groups are equal and no significant differences exist—would be rejected in favor of the alternative hypothesis (H1), which indicates that at least one group differs from the others. This significance level of 0.05 indicates a relatively low probability of observing such differences by random chance alone. Therefore, when the *p*-value is less than 0.05, it suggests that there is strong evidence to conclude that there are indeed significant differences among the groups being compared.

## 3. Results and Discussion

### 3.1. Preparation of THEESs

The studied THEESs formed a monophasic, homogeneous, clear, transparent and viscous liquid. No precipitate was observed at any time at room temperature.

### 3.2. Structural Study

Considering the ^1^H-NMR spectra for the studied THEESs, it can be observed that the values obtained for the integrals of each peak confirm that the stoichiometry of the system is adequate, indicating that no reaction has occurred. Additionally, the similarity of all the integrals can be seen, except for that of the alcohol groups in menthol and ibuprofen, due to the interactions in these eutectic mixtures are similar.

The spectra obtained for the different systems are gathered in the Supplementary Material (Figures S1 and S2). Furthermore, ^13^C NMR was performed on Ibu-M3, as no significant changes were observed in the spectrum compared to the other two eutectic mixtures. The spectrum clearly differentiates the various carbons. The Ibu-M3 spectra are gathered in the Supplementary Material (Figure S2).

On the other hand, a NOESY technique was performed on the three systems, where a significant number of negative signals were observed. The observed negative signals indicate the formation of a supramolecular network, which is a hallmark of ES, and DSC analysis further verified that this system meets the criteria to be classified as a DES (Figures S3–S5).

In addition, these negative signals are due to the high viscosity of these three THEESs. In fact, the high viscosity is also caused by the large interactions, including hydrogen bond interactions and, above all, van der Waals interactions. This is evident in the spectra, which reveal that nearly all protons within the molecules—aromatic hydrogens included—are involved in these interactions. Van der Waals interactions between ibuprofen molecules, menthol molecules or an ibuprofen molecule and menthol molecule help to stabilize the hydrogen bond interaction net.

Details of the NOESY and DOSY experiments are provided in the Supplementary Material (Figures S3–S5). These methods enabled the determination of diffusion coefficients for all three systems, as summarized in Table 2.

Results indicate that, as the composition of the eutectic liquid becomes more equimolar, diffusion decreases. Additionally, ibuprofen diffuses more slowly than menthol, although all the coefficients are very low, confirming that these are deep eutectic liquids [23].

### 3.3. Thermal Properties

Knowing the solid–liquid phase diagram (SLE) of a solvent is essential for its industrial implementation; furthermore, in the case of eutectic systems (ES), it is the only way to differentiate a DES/THEDES from an ES [24]. In Table 3, thermal properties for studied systems are presented.

For ibuprofen, the melting point obtained confirms the racemic form of this compound. The experimental value obtained agrees with that provided in the bibliography [26]. Regarding dl-menthol, the polymorphic behavior of this compound was evidenced during the experiment. At present, four different crystalline phases of this compound (α, β, γ *y* δ) have been reported, and the α phase is considered as the stable one. In our case, two different peaks were observed which have been related to the melting transitions of α phase and β phase taking into account the data presented by Stefja et al. [26]. Considering the eutectic mixtures, all of them present melting temperatures lower than room temperature which is a key factor in their industrial application. Regarding the experimental thermograms, only peaks due to the melting transition of *liquidus* fractions were observed for all mixtures. Bergua et al. reported a similar behavior in the thymol and L-menthol eutectic mixtures [27]. No presence of eutectic melting points is attributed to the strong interaction established between the compounds of the mixtures that hinders the rearrangement of the species and avoids the crystallization of eutectic fraction.

**Figure 1:**
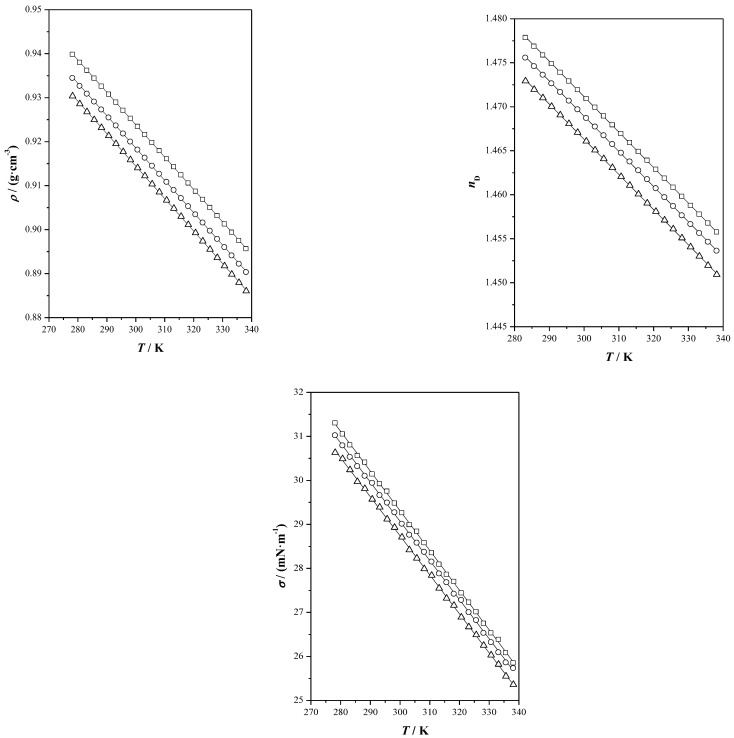
Effect of temperature *T* at *p* = 0.1 MPa, on the density, *ρ*, refractive index, *n*_D_, and surface tension, *σ*, of ibuprofen + dl-menthol systems: Ibu-M3 (☐), Ibu-M4 (◯), Ibu-M5 (△), (^____^) correlated values.

**Figure 2:**
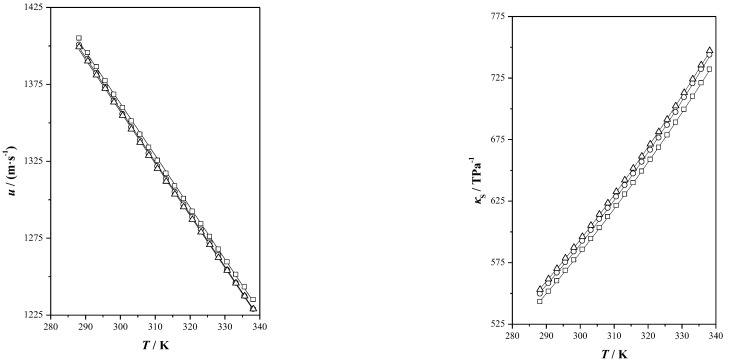
Speed of sound, *u*, and isentropic compressibility, *κ*_S_, of ibuprofen + dl-menthol systems as a function of temperature, *T*, at *p* = 0.1 MPa: Ibu-M3 (☐), Ibu-M4 (◯), Ibu-M5 (△), (^____^) correlated values.

**Figure 3:**
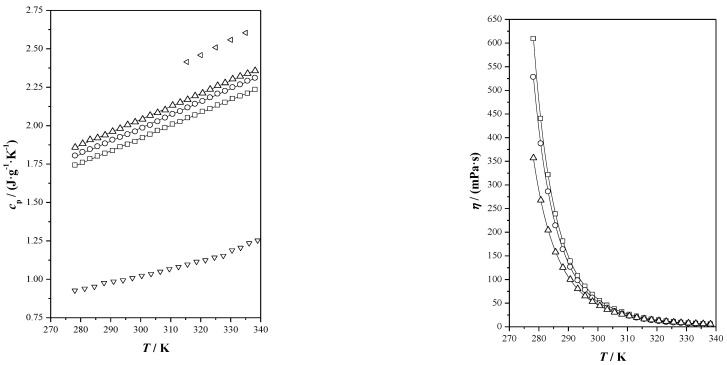
Variation in isobaric heat capacity, *c*_p_, and dynamic viscosity, *η*, of ibuprofen + dl-menthol systems with temperature, *T*, at *p* = 0.1 MPa: Ibu-M3 (☐), Ibu-M4 (◯), Ibu-M5 (△), Ibuprofen (▽), menthol (◁), (^____^) correlated values.

In this study, various properties were acquired and are presented in Table S1 of the Supplementary Material, encompassing both experimental measurements and calculated data for the investigated properties. Additionally, graphical representations of this information are provided in Figure 1, Figure 2 and Figure 3.

Furthermore, the way each property’s experimental results vary with temperature has been examined. Notably, a linear relationship with temperature was identified for several properties, including density, refractive index, surface tension, speed of sound and isobaric heat capacity. The experimental data were correlated using the following linear equation:

*Y* = *A*·*T* + *B* (5)

where *Y* denotes the relevant property, and *A* and *B* are parameters that can be adjusted.

The dynamic viscosity data were fitted using the Vogel–Fulcher–Tammann equation [28,29,30]:

(6) $$\eta =\eta_{0}\cdot exp\left[B/\left(T-T_{0}\right)\right]$$

with *η*_0_, *B* and *T*_0_ acting as parameters determined through the fitting parameters.

The fitting parameters derived from these models, along with the respective standard deviations, *S_yx_*, between the experimental and predicted values for each property, are presented in Table 4. The standard deviations, *S_yx_*, were determined according to the following mathematical formula:

(7) $$S_{yx}={\left(\frac{\sum {\left(y_{i}^{exp}-y_{i}^{cal}\right)}^{2}}{n-k}\right)}^{1/2}$$

where *k* represents the number of parameters estimated in the regression, and *n*-*k* corresponds to the degrees of freedom for the regression analysis.

Density is a very important property in the chemistry and pharmaceutical industries for a variety of reasons. It serves as a fundamental parameter for assessing not only container specifications and column diameters in industrial processes but also provides insights into chemical purity. Density offers valuable data concerning molecular interactions within chemical systems, and the molecular configuration in relation to the space occupied and the free volume [31]. In extraction processes, this parameter holds significance as it influences kinetic rates and the interplay between solvents and solid particles [32]. In the realm of pharmaceuticals, density assumes a critical role by furnishing essential insights into the physical properties of drug substances and their formulations. This data proves invaluable for optimizing drug dosages, ensuring physical stability and detecting counterfeit or substandard drugs [33].

Density is influenced by multiple factors, including the composition of the eutectic system and the nature of its constituent components [34].

Additionally, the molecular arrangement and interactions among the components within the mixture significantly contribute to this characteristic.

As can be observed in Figure 1, the density decreases as the temperature does, i.e., the systems are less dense at higher temperatures. Further analysis of the mixture shows that the density is higher for Ibu-M3, followed by Ibu-M4 and Ibu-M5. This is mainly due to the intermolecular interactions that occur between ibuprofen and menthol, primarily between the carboxylic group of ibuprofen and the alcohol of menthol.

The refractive index serves as a volumetric parameter that complements density measurements. In the context of pharmaceutical formulation, one significant application of this property is the assessment of drug substance purity and concentration [35]. Moreover, refractive index measurements can serve as a valuable tool for monitoring the physical stability of pharmaceutical formulations [36].

Changes in refractive index may indicate the emergence of crystals or other solid phases, which could affect the drug’s efficacy and safety. As expected, raising the temperature results in lower refractive index values, with refractive index displaying a linear correlation.

In this case, Figure 1 shows that the index values decrease as the proportion of menthol in the system increases, probably due to intermolecular interactions, as in the case of density. Higher values were found for Ibu-M3 followed by Ibu-M4 and Ibu-M5.

Surface tension is a critical parameter in several industrial processes. It significantly influences the design of equipment used in mixing and separation operations, as it is essential for determining fluid flow rates. The value of surface tension is governed by the strength of intermolecular interactions within the bulk of the liquid, rather than those present at the interface [31]. As the temperature increases, this property decreases because of the reduction in these bulk forces. In other words, a higher surface tension indicates stronger intermolecular forces. For the studied THEESs, higher values were obtained for Ibu-M3 followed by Ibu-M4 and Ibu-M5, thus, it can be deduced that the intermolecular interactions could be greater in Ibu-M3.

In Figure 2, we can observe the variations of both of these properties, with changing temperatures. The speed of sound is an important parameter in numerous fields, such as calculating constants for state equations, determining the bulk modulus, evaluating heat capacity and assessing the Joule–Thomson coefficient, among other uses [37]. This property offers valuable information regarding the compactness of molecular arrangements within a material. Higher speed of sound values are associated with more tightly packed molecules. In this study, the connection between speed of sound and temperature displays the expected inverse relationship: as the temperature increases, the measured speed of sound decreases. This trend aligns with established predictions and reflects the influence of temperature on molecular interactions. The speed of sound values are fairly consistent across the THEES samples analyzed, with Ibu-M3 exhibiting the highest values, followed by Ibu-M4 and Ibu-M5.

The internal structure of a DES can be analyzed using isentropic compressibility. When isentropic compressibility values are high, it suggests that the chemical structure within the mixture is less densely packed. Additionally, in cases such as the temperature rising, the isentropic compressibility values tend to increase. This information provides insights into the arrangement and behavior of molecules within the eutectic mixture, particularly how they respond to changes in temperature. For these mixtures, higher values were found for Ibu-M5 followed by Ibu-M4 and Ibu-M3.

Figure 3 displays the values for isobaric heat capacity, including the isobaric heat capacity of ibuprofen and menthol [26,38]. The isobaric heat capacities of the three systems studied are between menthol and ibuprofen. The isobaric heat capacity, which is closely associated with heat transfer processes, shows an upward trend with increasing temperature. Among the mixtures studied, Ibu-M5 exhibits the highest values, followed by Ibu-M4 and Ibu-M3. The additivity of isobaric heat capacity has been analyzed in the temperature range (315.65–335.65) K, in terms of average absolute deviation in *c*_p_, between the experimental values and those calculated using additivity. Presenting the following values for the systems studied, Ibu-M3: 0.0431 J·g^−1^·K^−1^, Ibu-M4: 0.0380 J·g^−1^·K^−1^, Ibu-M5: 0.0311 J·g^−1^·K^−1^, this deviation is smaller as the proportion of menthol is higher.

Lastly, the flow behavior of the THEES was determined using kinematic viscosity, *ν*. By incorporating experimentally obtained density values, the dynamic viscosity was derived. This property is an essential transport parameter, characterizing a fluid’s resistance to shear stress [39]. Importantly, viscosity is influenced by both mole composition and temperature. The establishment of a comprehensive viscosity database is crucial because viscosity plays a direct role in fluid flow calculations and the design of equipment. Access to such data during the design phase allows for the optimization of THEES compounds to achieve lower viscosity values, which can be advantageous in various industrial applications [40].

Most reported DESs or THEDESs exhibit elevated viscosity levels at ambient temperatures (η > 100 mPa·s). This elevated viscosity is mainly due to the extensive hydrogen bonding network formed between the constituents within these DESs. It is worth noting that DESs exhibit a broad spectrum of viscosity values [41].

For the THEESs under study, it was observed that the viscosity values were not as high as those of the other systems, with values at 298.5 K of 68.193 mPa·s, 61.406 mPa·s and 53.383 mPa·s for Ibu-M3, Ibu-M4 and Ibu-M5, respectively. The variation in viscosity can be influenced by multiple variables, such as temperature, water content, the chemical nature of the components and the molar ratio within the DES. These dependencies on viscosity have been the subject of previous research and are important considerations when working with DESs [41].

For the rheological analysis, the measured viscosity values were used to calculate the associated shear stress (*τ*). These results were subsequently fitted to the Herschel–Bulkley model, which describes the behavior of non-Newtonian fluids, as outlined below.

(8) $$\tau =\tau_{0}+kD^{n}$$

Within this model, the connection between shear stress and shear rate (D) is defined by the parameters $\tau_{0}$ (yield stress), *k* (consistency coefficient) and *n* (flow behavior index). The model also provided values for the coefficient of determination ($R^{2}=1-\;\sum {residual}^{2}/{\left(\bar{\tau }-\tau \right)}^{2}$) and standard deviation ($S=\;{\left(\sum {\left(residual\right)}^{2}/n-p\right)}^{2}$) where *n* represents the number of experimental measurements and *p* denotes the number of parameters included in the model.

Grasping the rheological properties of these systems is highly important in a range of disciplines, such as materials science, chemistry, pharmaceuticals and environmental research. This knowledge provides important information about the structure and stability of DESs or THEDESs. These systems stand out from traditional solvents, particularly due to their unique rheological features. Their viscosity can be easily tuned by altering the ratios of the components or by modifying the water content. This ability to adjust viscosity makes these systems exceptionally versatile, allowing them to be tailored for a wide variety of uses in different fields [42].

Furthermore, rheology is essential in the development of topical drug products, particularly in ensuring consistent quality during the formulation process [43]. This approach emphasizes the systematic design and optimization of drug formulations, ensuring their effectiveness and reliability. This study is crucial in enhancing processing efficiency and assisting formulators and end users in identifying pharmaceutical products tailored to their specific requirements. These measurements are applicable to a wide range of pharmaceutical products, including simple liquids, ointments, creams, pastes, suppositories, suspensions and various agents involved in dispersing, emulsifying and suspending colloidal substances [44].

**Figure 4:**
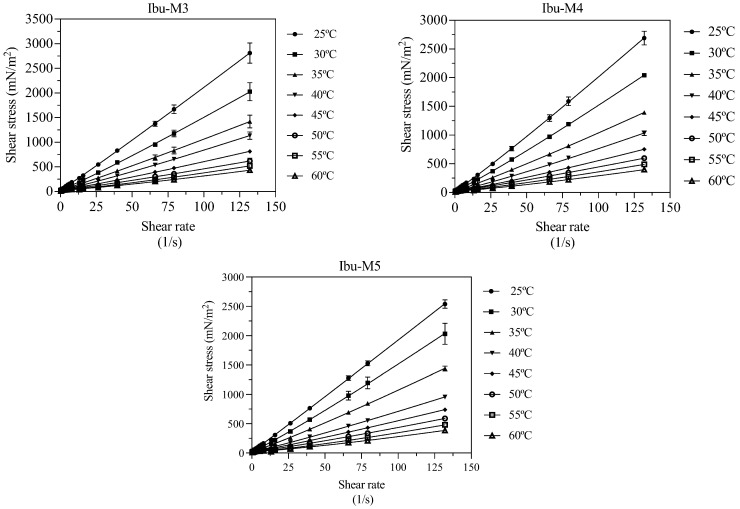
Rheological study, shear stress vs. shear rate, of the studied THEESs at the studied temperatures, (^____^) correlated values, Herschel–Bulkley model for non-Newtonian fluids (Equation (8)).

In the context of a specific study, the flow behavior of the investigated THEESs was evaluated. At a temperature of 25 °C, the study provided experimental data on the apparent viscosity of these THEESs, and the rheograms showcasing the flow behavior are illustrated in Figure 4. The analysis also involved the utilization of Equation (8) to determine adjusted parameters, which are documented in Table 5.

As expected, viscosity decreases with temperature. At each temperature, the three THEESs analyzed display very similar values, with IBU-M4 presenting slightly reduced figures compared to the others.

All the studied systems show non-Newtonian behavior. The deviation from linearity increases as the temperature rises in all cases, with n values being quite similar for the three THEESs at each of the studied temperatures, but higher than 1 in all cases, indicating dilatant behavior (viscosity increases as the shear stress does). This means that when these THEESs are subjected to higher shear forces, the fluids become thicker and more resistant to flow. Although this effect (thickening when shear force increases) is more pronounced as the temperature increases (*n* values increases with temperature), it is worth mentioning that for a given shear stress, viscosity will decrease with temperature.

The observation that the flow index n increases with temperature in shear-thickening or dilatant fluids can be explained by several mechanisms supported by the existing literature. One contributing factor is that an increase in shear rate causes particles within the suspension to move closer together, enhancing resistance to flow due to microstructural rearrangement. This increased resistance is characteristic of dilatant behavior and has been reported in systems such as passion fruit seed oil, where viscosity decreases with increasing temperature, yet resistance to flow intensifies with higher shear rates [45]. Additionally, as the temperature rises, the effectiveness of lubrication provided by solid particles tends to decrease. This reduced lubrication, coupled with the possible interlocking of solid grains under shear, can lead to a rise in the apparent viscosity and an increase in the flow index. Similar effects were documented in melter feed systems during high-temperature processing, where particle interactions and grain interlocking under applied force contributed to rheological complexity [46]. These findings align with the experimental increase in n observed in this study, suggesting that the dilatant response intensifies with thermal and mechanical conditions that promote particle contact and structural rigidity.

Furthermore, although of small magnitude, there is a threshold shear stress force, *τ*_0_, necessary for the fluid to begin doing so. This threshold shear stress force is slightly higher for Ibu-M3 followed by Ibu-M4, with the smaller values being for THEES’s Ibu-M5. However, the effect of the temperature is not very intense, and threshold shear stress force values remain almost constant when temperature increases.

### 3.4. Cytotoxicity Results

This research was conducted using two different cell lines to assess the safety of these THEESs for potential use in pharmaceutical formulations. The chosen cell lines included HaCaT (a keratinocyte line), which helps provide an initial indication of whether these THEESs could be suitable for topical drug administration, and HepG2 (a hepatic cell line), since ibuprofen undergoes metabolism in the liver.

#### 3.4.1. Prestoblue Assay Results

PrestoBlue is a cell viability assay reagent that relies on resazurin, a blue, non-toxic and weakly fluorescent compound capable of entering cells. When added to a cell culture, metabolically active (living) cells reduce resazurin to resorufin, which is red and highly fluorescent. This chemical transformation occurs only in viable cells, causing a visible color change and increased fluorescence. By measuring these changes, researchers can quantitatively determine the proportion of living cells and assess potential cytotoxic effects of tested substances. This method offers a straightforward and sensitive approach to monitor cell health and viability in various experimental settings [47].

**Figure 5:**
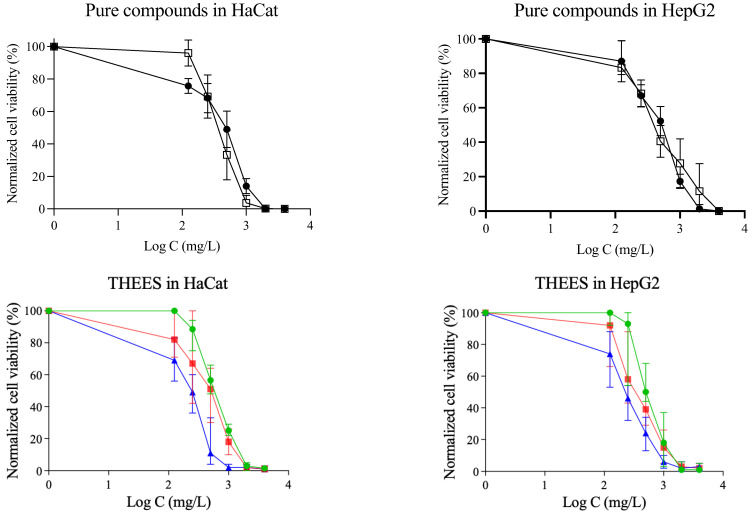
Dose–response curves in studied cell lines for: ibuprofen (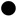), menthol (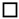), Ibu-M3 (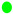), Ibu-M4 (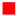) and Ibu-M5(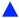).

This assay evaluates the capacity of cells to transform a specific salt into a different compound. A greater conversion rate indicates higher cellular metabolic activity, which in turn reflects a larger number of viable cells. Figure 5 presents the dose–response curves for the THEESs tested in both HaCaT and HepG2 cell lines. In every case, cell viability declines as the concentration of the THEESs increases. The pattern of toxicity is consistent across both cell types: as the menthol content rises, so does the toxicity. Among the systems tested, Ibu-M5 is the most toxic, followed by Ibu-M4 and Ibu-M3. The EC_50_ values for both cell lines are summarized in Table 6.

It is worth noting that there are similar studies, such as the one published by Pereira et al. where they tested a THEDES formed by ibuprofen and menthol in a 1:3 ratio on the Caco-2 cell line (human colorrectal adenocarcinoma), obtaining values of ibuprofen (2.89 mM ± 0.06 or 596.2 mg/L [13]) menthol (5.09 mM ± 0.73 or 795.4 mg/L [45]) and 8.92 mM ± 1.39 or 1505.5 mg/L for the Ibu-M3 system [12,45]. These values differ from those obtained in this study because the cell lines, and therefore their behavior, are different. However, the trend remains the same, i.e., the formed THEESs are less toxic than the pure compounds.

Furthermore, Table S2 shows the *p* values from the ANOVA statistical analysis that was performed to test for significant differences between the pure compounds and the studied systems. As can be seen, all *p* values are less than 0.05; in fact, all except those for ibuprofen and menthol are below 0.0001. This indicates significant differences between the pure compounds tested and the analyzed systems.

#### 3.4.2. Crystal Violet Assay Results

This assay is a quick and adaptable technique used to evaluate cell viability after exposure to different factors, such as chemicals or toxic substances. In this method, adherent cells that die will detach from the surface of the culture plate. This detachment provides an indirect way to measure cell death in the culture and helps to assess changes in cell growth after treatment with specific agents. The procedure involves staining the cells with crystal violet dye, which attaches to cellular proteins and DNA. As cells die and detach, the amount of dye retained on the plate decreases, resulting in lighter staining. This decrease in staining intensity reflects the number of viable cells remaining and shows how the tested stimulus affects cell growth and survival [49].

**Figure 6:**
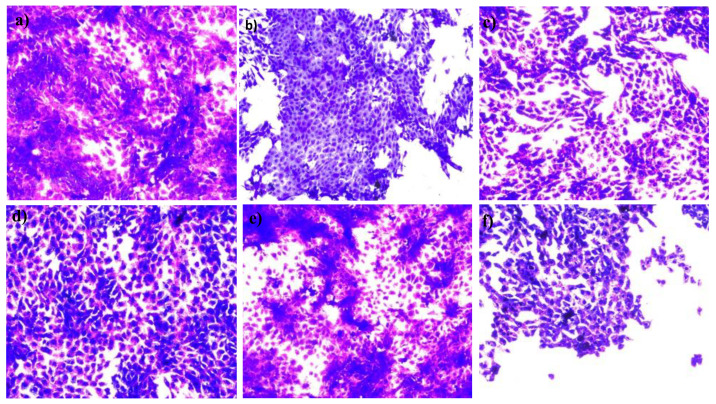
Crystal violet assay at 24 h after the exposure of 250 mg/L at 20× magnification of pure compounds and THEESs with the HaCaT cell line. (**a**) Control, (**b**) ibuprofen, (**c**) menthol, (**d**) Ibu-M3, (**e**) Ibu-M4, (**f**) Ibu-M5.

**Figure 7:**
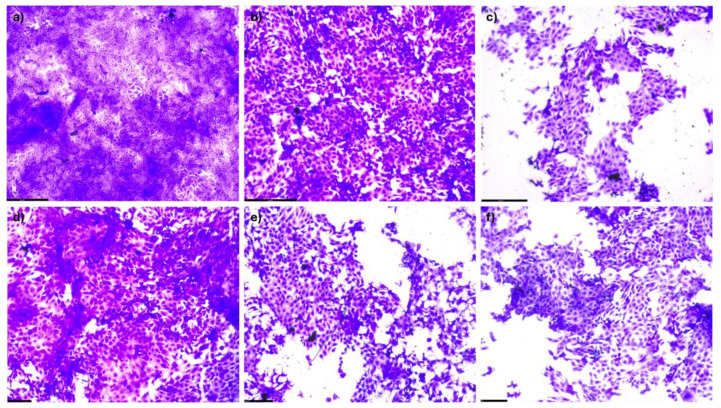
Crystal violet assay at 24 h after the exposure of 250 mg/L at 20× magnification of pure compounds and THEESs with the HepG2 cell line. (**a**) Control, (**b**) ibuprofen, (**c**) menthol, (**d**) Ibu-M3, (**e**) Ibu-M4, (**f**) Ibu-M5.

The test was designed to determine the toxicity of the THEESs being studied by utilizing both descriptive analysis and numerical data. In the qualitative aspect, a series of images were captured using a light microscope to observe the cellular effects following exposure to THEESs. Figure 6 and Figure 7 display multiple photographs taken at 20X magnification, illustrating the comparison between the control sample and the samples treated with THEESs at 250 mg/L concentration for the HaCaT and HepG2 cell lines after a 24 h exposure period. This visual examination allowed for the evaluation of cellular responses to THEES exposure.

Analyzing the photographs, for both cell lines, ibuprofen and menthol are more cytotoxic than the systems analyzed. Furthermore, when analyzing the systems at 250 mg/L, the cell concentration decreases as the proportion of menthol increases. This may be because menthol has a lower EC_50_ than ibuprofen, making a DES with more menthol more toxic.

**Figure 8:**
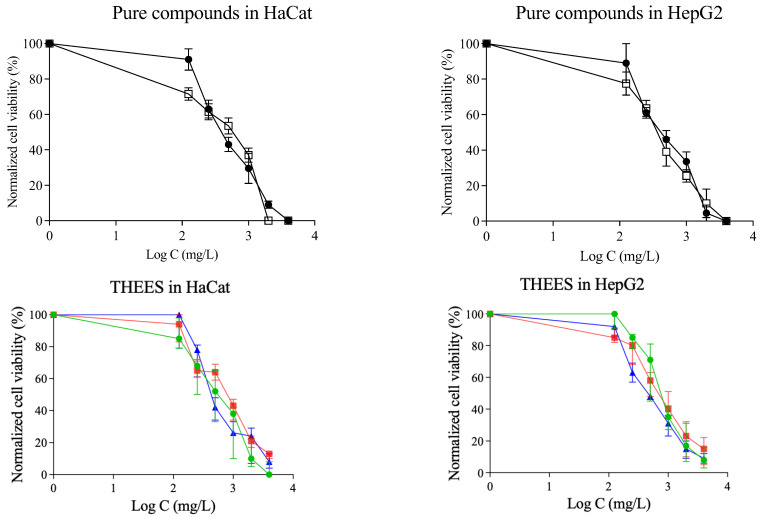
Dose–response curves in HaCat and HepG2 cell lines for crystal violet assay: ibuprofen (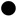), menthol (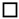), Ibu-M3 (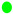), Ibu-M4 (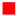); and Ibu-M5 (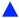).

Figure 8 displays the dose–response curves for HaCaT and HepG2 cells after 24 h. Both the qualitative and quantitative results follow the same pattern: cell viability declines as the concentration of menthol is increased, with the Ibu-M5 system exhibiting the highest toxicity in all cases. In qualitative studies, the trend is the same as in Prestoblue. However, in quantitative studies, it is observed that the trend is the same for the HepG2 line, but not so clear for HaCat.

### 3.5. BCA Protein Assay Kit

The bicinchoninic acid (BCA) assay enables reliable measurement of protein concentration. This straightforward technique is compatible with certain detergents and is typically conducted in a microplate format [50,51,52]. The assay’s color formation relies on a two-step process. Initially, a protein in an alkaline environment catalyzes the reduction of Cu^2+^ ions to Cu^+^ ions through the biuret reaction. Subsequently, BCA interacts with Cu+ ions, leading to the formation of a distinctive purple-colored complex. This complex’s absorbance can be precisely measured at 562 nm [51].

**Figure 9:**
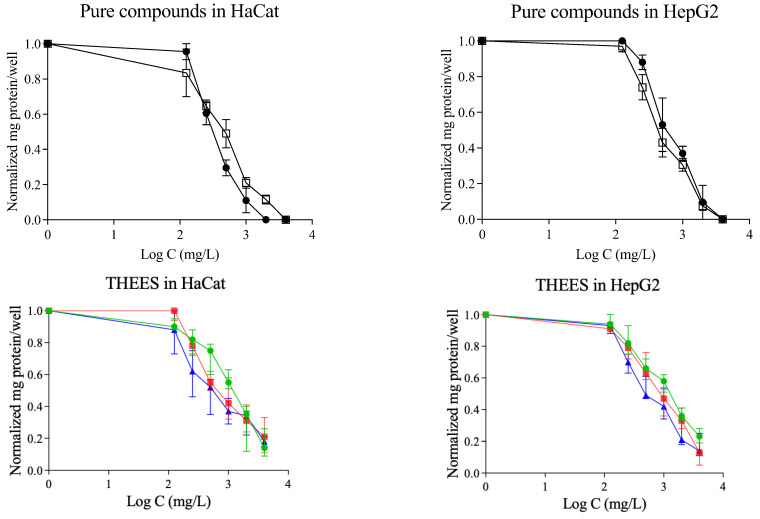
Dose–response curves in HaCat and HepG2 cell lines for BCA assay: ibuprofen (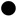), menthol (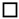), Ibu-M3 (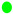), Ibu-M4 (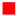) and Ibu-M5 (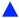).

Figure 9 presents the cytotoxicity observed 24 h after THEES exposure. The data demonstrate that the toxic effects are dependent on the dose and align with the findings from the PrestoBlue assay. As the concentration of the tested compounds rises, the normalized protein content declines. Both the cell viability and protein quantification graphs display comparable trends; the decrease in normalized fluorescence seen in the PrestoBlue assay corresponds to the drop in normalized absorbance measured by the BCA method.

## 4. Conclusions

In this paper, three THEESs formed by ibuprofen and menthol at different molar ratios have been prepared. Additionally, a physicochemical and cytotoxicological study has been carried out. The prepared systems were monophasic, homogeneous, clear, transparent and viscous liquids. From a physicochemical point of view, it has been observed that these systems, akin to many others, exhibit a temperature-dependent relationship with the analyzed property. Specifically, for density, refractive index, surface tension, speed of sound and isobaric molar heat capacity, the relationship is inversely proportional. In the case of viscosity, it follows a behavior that conforms to the Vogel–Fulcher–Tammann equation. Regarding rheological study, it has been noted that these systems exhibit non-Newtonian behavior. Understanding all these properties is crucial for pharmaceutical formulation at an industrial level. At the cytotoxic level, it was observed that, in HaCaT cells, the Ibu-M3 and Ibu-M4 mixtures exhibited lower cytotoxicity compared to pure ibuprofen or menthol. In contrast, the Ibu-M5 system demonstrated significantly higher cytotoxicity than the individual compounds. In the case of HepG2 cells, only the Ibu-M3 system showed reduced cytotoxicity relative to the pure substances, whereas both Ibu-M4 and Ibu-M5 systems were notably more cytotoxic than pure ibuprofen and menthol.

## Figures and Tables

**Table 1:** Abbreviation, molar ratio and molecular weight of three studied THEESs.

THEES	Abbreviation	Molar Ratio	Molar Mass (g/mol)
Ibuprofen/menthol	Ibu-M3	1:3	168.78
	Ibu-M4	1:4	166.27
	Ibu-M5	1:5	164.61

**Table 2:** The diffusion coefficients, *D*, of the 3 THEESs at 298 K. Aliphatic hydrogens of ibuprofen (I), aliphatic hydrogens of menthol (M) and hydroxyl hydrogen of ibuprofen.

THEES	10^11^ D_I_/m^2^·s^−1^	10^11^ D_M_/m^2^·s^−1^	10^11^ D_OH_/m^2^·s^−1^
Ibu-M3	1.174	1.843	1.868
Ibu-M4	1.271	2.002	1.953
Ibu-M5	1.279	2.041	2.032

**Table 3:** Information of the prepared THEESs and thermal properties (melting temperature, *T_m_*, and melting enthalpy, Δ*_m_H*). α and β correspond to two of the crystalline phases of menthol.

Compound/System	Abbreviation	Composition (Molar Ratio)	Molar Mass(g/mol)	*T_m,exp_* (K)	*T_m,lit_* (K)
Ibuprofen	-	-	206.29	347.33	344.15 [25]
DL-Menthol	-	-	156.27	α = 307.44	α = 307.44 [26]
				β = 300.26	β = 300.26 [26]
Ibuprofen/menthol	Ibu-M3	1:3	168.78	253.00	**-**
	Ibu-M4	1:4	166.27	282.61	**-**
	Ibu-M5	1:5	164.61	282.41	**-**

**Table 6:** Values of EC_50_ at 24 in HaCaT and HepG2 cell lines for studied THEESs and bibliographic data.

THDES	HaCaT (mg/L)	HepG2 (mg/L)	Caco-2 (mg/L)
Ibuprofen	388 ± 8	435 ± 7	596.2 [13]
Menthol	361 ± 8	419 ± 9	795.4 [48]
Ibu-M3	579 ± 5	530 ± 8	1505.5 [12,48]
Ibu-M4	427 ± 11	352 ± 8	-
Ibu-M5	218 ± 6	228 ± 6	-

**Table 4:** Fitting parameters (*A*, *B*, *C*) along with the standard deviation *S*, for the measured properties.

THEES	Property	*A*	*B*	*C*	*S*
Ibu-M3	*ρ*/(g·cm^−3^)	−0.000736	1.14476		0.0001
	*u*/(m·s^−1^)	−3.302	2353.35		1.96
	*n*_D_	−0.000402	1.591809		0.0001
	*σ*/(mN·m^−1^)	−0.0902	56.36		0.03
	*c*_p_/(J·g^−1^·K^−1^)	−0.5622	0.00827		0.0030
	*η* ^a^/(mPa·s)	0.0097	894.7	197.17	0.58
Ibu-M4	*ρ*/(g·cm^−3^)	−0.000735	1.13917		0.0002
	*u*/(m·s^−1^)	−3.400	2387.67		0.74
	*n*_D_	−0.000399	1.588761		0.0002
	*σ*/(mN·m^−1^)	−0.0890	55.76		0.03
	*c*_p_/(J·g^−1^·K^−1^)	−0.5677	0.00851		0.0043
	*η*/(mPa·s)	0.0016	1253.3	179.66	0.60
Ibu-M5	*ρ*/(g·cm^−3^)	−0.000738	1.13598		0.0002
	*u*/(m·s^−1^)	−3.295	2349.22		5.09
	*n*_D_	−0.000404	1.586233		0.0001
	*σ*/(mN·m^−1^)	−0.0887	55.34		0.03
	*c*_p_/(J·g^−1^·K^−1^)	−0.47171	0.00837		0.0039
	*η*/(mPa·s)	0.0174	839.1	193.64	0.41

^a^ *A* = *η*_0_; *C* = *T*_0_.

**Table 5:** Adjusted parameters, *τ*_0_, *k* and *n* (Equation (8)) with their corresponding coefficient of determination, R^2^ and standard deviation, s, at the studied temperatures.

T (K)	298.15	303.15	308.15	313.15	318.15	323.15	328.15	333.15
Ibu-M3								
*τ*_0_ (mN/m^2^)	29.94	32.67	30.02	33.26	28.10	27.44	27.15	26.95
*k* (g/s·m)	17.31	10.06	7.523	4.599	3.392	2.478	1.287	1.052
*n*	1.040	1.083	1.069	1.122	1.116	1.121	1.218	1.220
R^2^	0.9959	0.9936	0.9921	0.9955	0.9975	0.9891	0.9943	0.9933
s	47.82	42.91	33.23	19.87	10.49	16.53	9.805	8.809
Ibu-M4								
*τ*_0_ (mN/m^2^)	26.29	28.51	27.09	25.64	26.75	25.60	23.98	22.38
*k* (g/s·m)	14.72	9.726	6.534	3.98	2.631	2.355	1.458	0.998
*n*	1.065	1.092	1.094	1.134	1.151	1.125	1.180	1.214
R^2^	0.9978	0.9993	0.999	0.997	0.9965	0.995	0.996	0.9929
s	33.70	13.85	11.32	14.83	11.36	10.76	7.783	8.365
Ibu-M5								
*τ*_0_ (mN/m^2^)	20.90	25.53	22.91	24.01	22.63	23.31	22.97	22.97
*k* (g/s·m)	17.50	10.04	6.872	4.217	3.223	2.090	1.040	0.8226
*n*	1.018	1.086	1.092	1.106	1.107	1.148	1.247	1.247
R^2^	0.9989	0.993	0.998	0.9982	0.9967	0.9961	0.995	0.9928
s	22.79	45.40	16.97	10.55	11.13	9.413	8.546	8.1240

## Data Availability

All data are included in the text.

## Supplementary Materials

The following supporting information can be downloaded at: https://www.mdpi.com/article/10.3390/pharmaceutics17080979/s1, Figure S1: Spectra of the ^1^H-RMN of the 3 THEES Ibuprofen:Menthol; Figure S2. Spectra of the ^13^C-RMN of the Ibuprofen:Menthol (1:3); Figure S3. Spectra of the NOESY and DOSY of Ibuprofen:Menthol (1:3); Figure S4. Spectra of the NOESY and DOSY of Ibuprofen:Menthol (1:4); Figure S5. Spectra of the NOESY and DOSY of Ibuprofen:Menthol (1:5); Table S1. Thermophysical properties and some derived properties of ibuprofen + dl-menthol systems as a function of temperature; Table S2. *p* values for the toxicity (EC50) statistical analysis for pure compounds and studied THEES.
