# Supplementary material for: Exploring Ibuprofen–Menthol Eutectic Systems: Physicochemical Properties and Cytotoxicity for Pharmaceutical Applications
Source: Pharmaceutics. 2025 Jul 29;17(8):979. doi: 10.3390/pharmaceutics17080979 (PMC12389556; doi:10.3390/pharmaceutics17080979)
Supplement: Supplementary file 1 [file pharmaceutics-17-00979-s001.zip › pharmaceutics-3739449-supplementary.pdf]

Supplementary information

Exploring Ibuprofen–Menthol Eutectic Systems: Physicochemical Properties and Cytotoxicity for Pharmaceutical Applications

Álvaro Werner [1], Estefanía Zuriaga [1], Marina Sanz [1], Fernando Bergua [2], Beatriz Giner [1], Carlos Lafuente [2] and Laura Lomba [1,*]

[1] Facultad de Ciencias de la Salud, Universidad San Jorge, Campus Universitario, Autov. A23 km 299, Villanueva de Gállego, 50830 Zaragoza, Spain; awerner@usj.es (Á.W.); ezuriaga@usj.es (E.Z.); bginer@usj.es (B.G.)

[2] Departamento de Química Física, Facultad de Ciencias, Universidad de Zaragoza, Zaragoza, 50009 Zaragoza, Spain; ferber@posta.unizar.es (F.B.); celadi@unizar.es (C.L.)

* Correspondence: llomba@usj.es

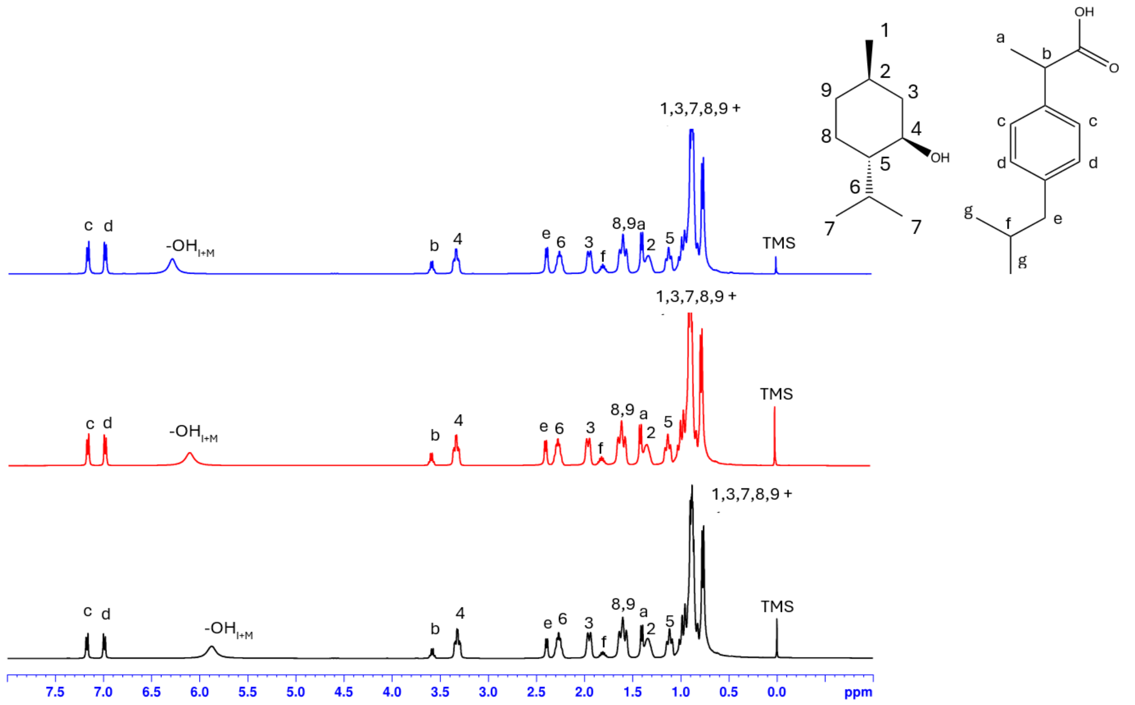

Figure S1. Spectra of the ^{1}H-RMN of the 3 THEES Ibuprofen:Menthol.

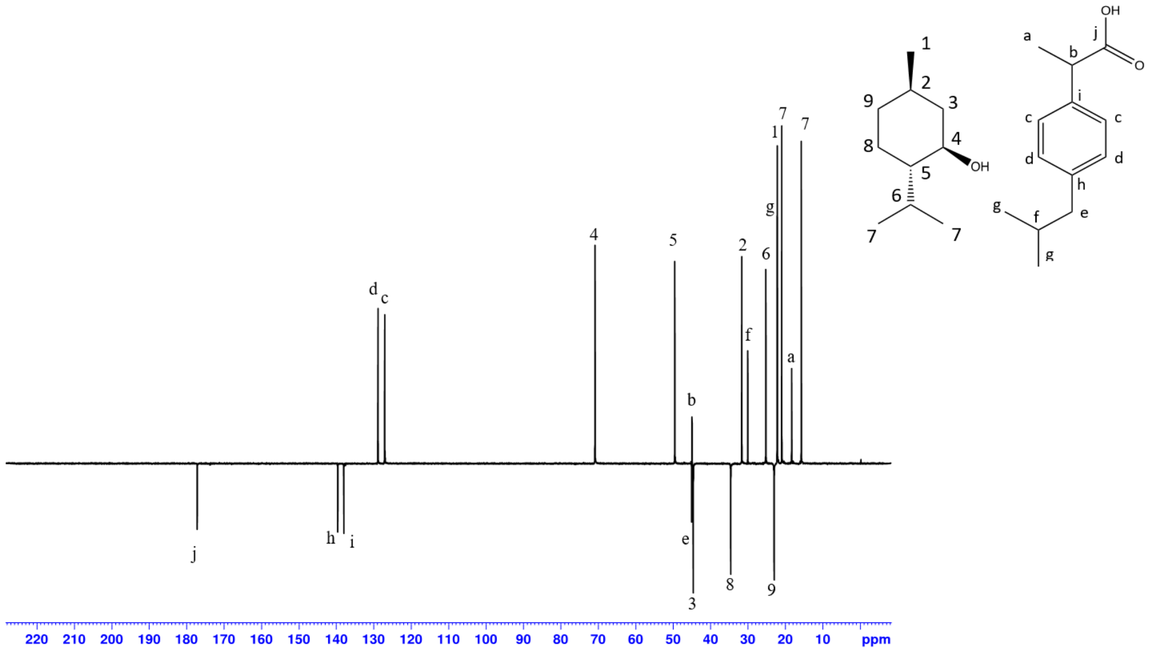

Figure S2. Spectra of the ^{13}C-RMN of the Ibuprofen:Menthol (1:3).

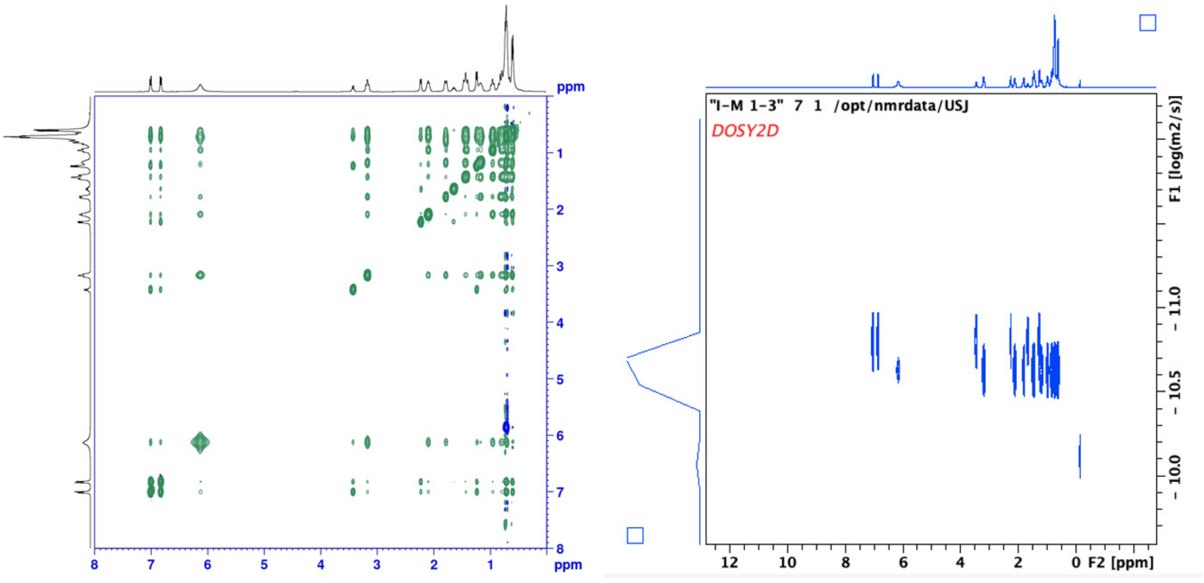

Figure S3. Spectra of the NOESY and DOSY of Ibuprofen:Menthol (1:3).

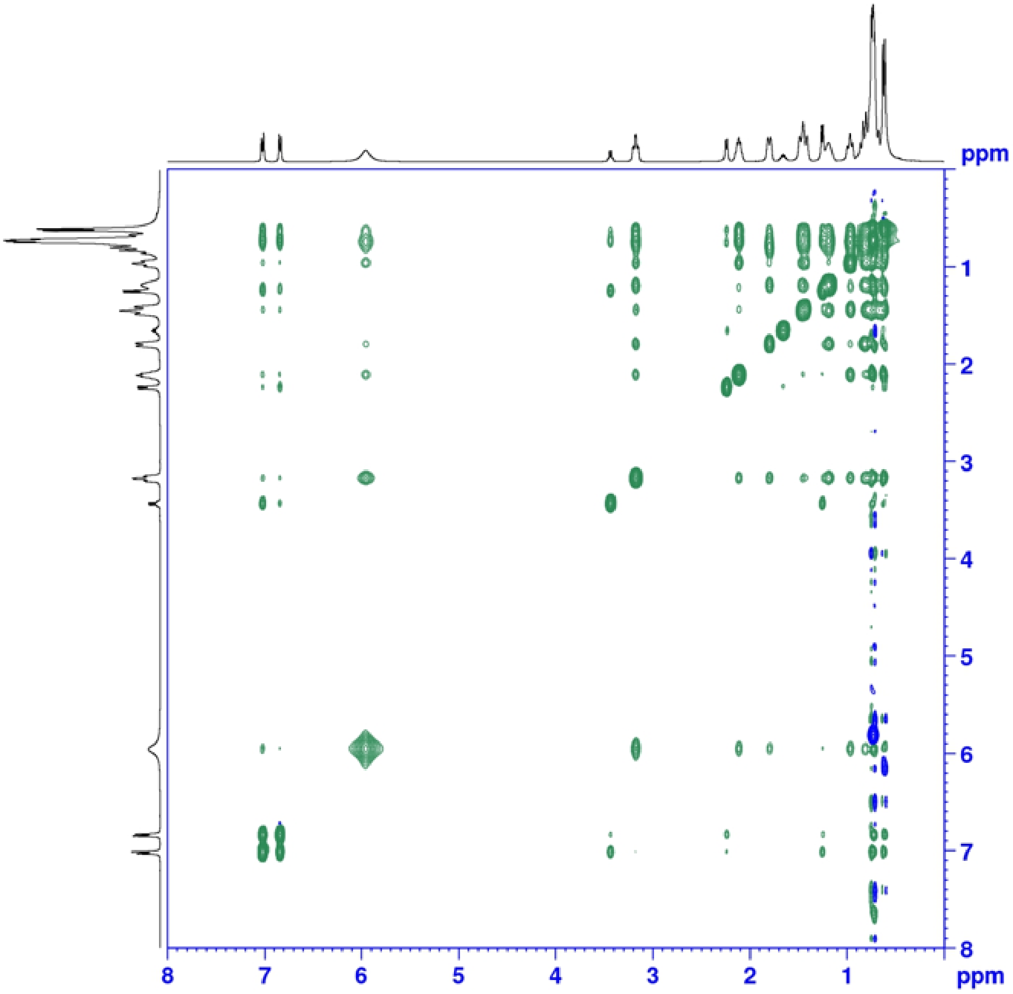

Figure S4. Spectra of the NOESY and DOSY of Ibuprofen:Menthol (1:4).

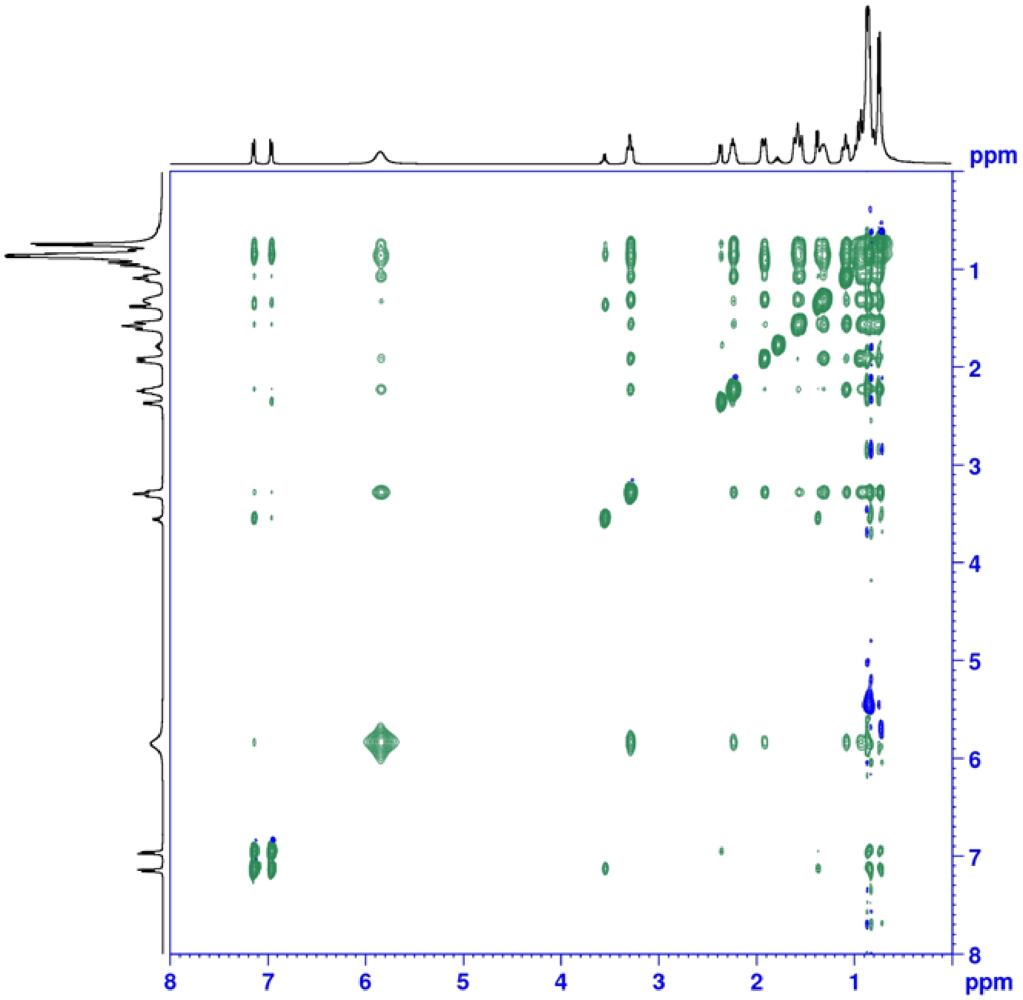

Figure S5. Spectra of the NOESY and DOSY of Ibuprofen:Menthol (1:5).

Table S1. Thermophysical properties and some derived properties of ibuprofen + dl-menthol systems as a function of temperature, T, at atmospheric pressure, p = 0.10 MPa: density, ρ, speed of sound, u, isentropic compressibility, κ_S, refractive index, n_D, surface tension, σ, isobaric heat capacity, c_p, kinematic viscosity, ν, and dynamic viscosity, η.[a]

T / K	ρ / (g·cm^{-3})	u / (m·s^{-1})	κ_S / (TPa^{-1})	n_D	σ / (mN·m^{-1})	c_p / (J·g^{-1}·K^{-1})	ν / (mm·s^{-1})	η / (mPa·s)
Ibuprofen + dl-menthol (1:3)								
278.15	0.93799				31.30	1.742	648.13	609.12
280.65	0.93619				31.05	1.760	469.40	440.29
283.15	0.93439			1.477849	30.80	1.784	343.09	321.19
285.65	0.93257			1.476852	30.56	1.801	255.25	238.51
288.15	0.93075	1404.96	543.24	1.475863	30.41	1.819	193.84	180.77
290.65	0.92892	1395.55	551.67	1.474875	30.14	1.837	148.98	138.66
293.15	0.92708	1386.35	560.11	1.473893	29.92	1.861	116.20	107.94
295.65	0.92525	1377.34	568.59	1.472894	29.75	1.878	92.036	85.325
298.15	0.92342	1368.49	577.11	1.471914	29.48	1.896	73.703	68.193
300.65	0.92158	1359.75	585.71	1.470917	29.26	1.920	59.615	55.050
303.15	0.91975	1351.12	594.40	1.469916	28.99	1.943	49.139	45.286
305.65	0.91792	1342.47	603.28	1.468929	28.84	1.967	40.679	37.415

308.15	0.91608	1334.01	612.18	1.467926	28.58	1.985	33.936	31.151
310.65	0.91425	1325.54	621.27	1.466921	28.35	2.009	28.436	26.050
313.15	0.91240	1317.08	630.54	1.465915	28.09	2.026	24.117	22.049
315.65	0.91056	1308.88	639.76	1.464902	27.86	2.050	20.455	18.663
318.15	0.90870	1300.65	649.19	1.463897	27.70	2.068	17.693	16.111
320.65	0.93799	1292.45	658.80	1.462870	27.44	2.092	15.256	13.863
323.15	0.90685	1284.31	668.54	1.461856	27.23	2.109	13.246	12.012

T / K	ρ / (g·cm^{-3})	u / (m·s^{-1})	κ_S / (TPa^{-1})	n_D	σ / (mN·m^{-1})	c_p / (J·g^{-1}·K^{-1})	ν / (mm·s^{-1})	η / (mPa·s)
325.65	0.90498	1276.00	678.67	1.460830	27.01	2.133	11.454	10.366
328.15	0.90312	1267.73	688.97	1.459788	26.75	2.151	10.048	9.0747
330.65	0.90125	1259.47	699.49	1.458766	26.53	2.175	8.8840	8.0067
333.15	0.89936	1251.34	710.09	1.457765	26.38	2.192	7.8817	7.0885
335.65	0.89749	1243.21	720.91	1.456762	26.08	2.210	7.0214	6.3016
338.15	0.89560	1235.03	732.03	1.455738	25.85	2.234	6.2847	5.6286
Ibuprofen + dl-menthol (1:4)								
278.15	0.93444				31.02	1.804	565.18	528.13
280.65	0.93264				30.79	1.828	415.47	387.48
283.15	0.93086			1.475561	30.53	1.846	307.06	285.83
285.65	0.92907			1.474595	30.32	1.864	230.40	214.05
288.15	0.92727	1400.68	549.69	1.473615	30.10	1.883	176.41	163.58
290.65	0.92545	1391.34	558.19	1.472637	29.94	1.907	136.66	126.47
293.15	0.92363	1382.20	566.71	1.471654	29.66	1.925	106.02	97.927
295.65	0.92179	1373.23	575.28	1.470666	29.49	1.943	84.265	77.675
298.15	0.91997	1364.36	583.94	1.469684	29.27	1.961	66.748	61.406
300.65	0.91814	1355.66	592.64	1.468696	29.01	1.985	53.870	49.460
303.15	0.91631	1347.08	601.41	1.467720	28.76	2.003	43.629	39.978
305.65	0.91448	1338.49	610.37	1.466726	28.58	2.027	35.996	32.917
308.15	0.91265	1330.06	619.37	1.465739	28.37	2.051	30.105	27.476
310.65	0.91081	1321.65	628.55	1.464751	28.15	2.075	24.988	22.760
313.15	0.90898	1313.29	637.86	1.463755	27.88	2.093	20.950	19.043
315.65	0.90713	1305.02	647.29	1.462753	27.68	2.117	17.703	16.059
318.15	0.90528	1296.82	656.84	1.461746	27.42	2.141	14.800	13.398
320.65	0.90343	1288.64	666.56	1.460717	27.28	2.159	12.771	11.538
323.15	0.90157	1280.52	676.44	1.459698	27.00	2.183	10.818	9.7528
325.65	0.89971	1272.16	686.77	1.458690	26.82	2.207	9.5118	8.5579
328.15	0.89784	1263.88	697.25	1.457633	26.53	2.225	8.2448	7.4025
330.65	0.89597	1254.38	709.33	1.456644	26.32	2.249	7.1550	6.4107
333.15	0.89409	1245.88	720.55	1.455625	26.09	2.267	6.2453	5.5839
335.65	0.89220	1237.38	732.03	1.454634	25.86	2.291	5.4990	4.9062
338.15	0.89031	1228.88	743.77	1.453615	25.73	2.310	4.8483	4.3165
Ibuprofen + dl-menthol (1:5)								
278.15	0.93036				30.63	1.859	383.79	357.06
280.65	0.92857				30.49	1.883	288.24	267.65
283.15	0.92678			1.472920	30.24	1.908	220.49	204.35
285.65	0.92498			1.471966	29.97	1.920	170.62	157.82
288.15	0.92317	1399.32	553.20	1.471005	29.81	1.938	135.78	125.35
290.65	0.92135	1390.05	561.71	1.470032	29.57	1.962	108.61	100.07
293.15	0.91952	1381.09	570.16	1.469058	29.39	1.981	87.525	80.481
295.65	0.91769	1372.24	578.69	1.468074	29.12	2.005	70.854	65.022
298.15	0.91586	1363.42	587.37	1.467072	28.93	2.023	58.287	53.383

300.65	0.91404	1354.73	596.11	1.466082	28.71	2.041	48.558	44.384
303.15	0.91220	1345.82	605.25	1.465080	28.42	2.066	39.918	36.414
305.65	0.91037	1337.21	614.30	1.464082	28.23	2.084	33.839	30.806
308.15	0.90853	1328.72	623.44	1.463075	27.99	2.102	29.192	26.522
310.65	0.90668	1320.29	632.71	1.462055	27.84	2.132	25.152	22.805
313.15	0.90483	1311.91	642.13	1.461060	27.55	2.151	21.630	19.571
315.65	0.90298	1303.60	651.68	1.460066	27.32	2.169	18.392	16.608
318.15	0.90112	1295.32	661.40	1.459044	27.16	2.193	16.044	14.458
320.65	0.89926	1287.09	671.27	1.458091	26.89	2.211	14.074	12.656
323.15	0.89738	1278.94	681.28	1.457107	26.67	2.236	12.544	11.257
325.65	0.89551	1270.80	691.47	1.456104	26.49	2.260	11.020	9.8688
328.15	0.89363	1262.34	702.25	1.455085	26.25	2.278	9.8057	8.7627
330.65	0.89174	1254.04	713.08	1.454061	26.03	2.303	8.8194	7.8646
333.15	0.88985	1245.76	724.13	1.453008	25.82	2.321	7.9408	7.0661
335.65	0.88794	1237.38	735.55	1.451955	25.55	2.339	7.1346	6.3351
338.15	0.88603	1228.98	747.24	1.450920	25.36	2.357	6.5156	5.7730

[a] Standard uncertainties u are $u(T)$ = 0.005 K for density and speed of sound and $u(T)$ = 0.01 K for the rest of properties, $u(p)$ = 1 kPa, and the combined expanded uncertainties U_c are $U_c(\rho) = 1{\cdot}10^{-4}$ g·cm^{-3}, $U_c(u) = 0.5$ m·s^{-1}, $U_c(n_D) = 5{\cdot}10^{-5}$, $U_c(u) = 0.5$ mN·m^{-1}, $U_c(c_P) = 1{\cdot}10^{-3}$ J·g^{-1}·K^{-1}, $U_c(\nu) = 5$ mm·s^{-1} with 0.95 level of confidence ($k = 2$).

Table S2. p values for the toxicity (EC_{50}) statistical analysis for pure compounds and studied THEES.

	**Ibuprofen**	**Menthol**	**Ibu-M3**	**Ibu-M4**	**Ibu-M5**
Ibuprofen	-	-	-	-	-
Menthol	0.0003	-	-	-	-
Ibu-M3	<0.0001	<0.0001	-	-	-
Ibu-M4	<0.0001	<0.0001	<0.0001	-	-
Ibu-M5	<0.0001	<0.0001	<0.0001	<0.0001	-
